# A Rare Case of Parvovirus B19 Infection Manifesting as Chronic Aplastic Anemia and Neutropenia in a Human Immunodeficiency Virus-Infected Patient

**DOI:** 10.7759/cureus.12174

**Published:** 2020-12-19

**Authors:** Elona Shehi, Haider Ghazanfar, Ked Fortuzi, Efrain Gonzalez, Cosmina Zeana

**Affiliations:** 1 Gastroenterology, BronxCare Health System, Bronx, USA; 2 Internal Medicine, BronxCare Health System, Bronx, USA; 3 Infectious Disease, BronxCare Health System, Bronx, USA

**Keywords:** parvovirus b19, immunocompromised, neutropenia, immunoglobulin, aplastic anemia

## Abstract

Parvovirus B19 (PVB19) is a deoxyribonucleic acid (DNA) virus, the only member of the Parvoviridae, which has a direct cytopathic effect on erythroid progenitor cells, resulting in an arrest of hematopoiesis and subsequent anemia. Less frequently, neutropenia and thrombocytopenia have been reported with the PVB19 infection. We report a rare case of chronic neutropenia due to PVB19 in a human immunodeficiency virus (HIV) patient. A 51-year-old male with a medical history of HIV presented to the Emergency Department (ED) with complaints of generalized weakness. His laboratory tests were significant for severe anemia and new neutropenia. PVB19 DNA by polymerase chain reaction (PCR) was positive. PVB19 immunoglobulin M (IgM) and IgG were reported negative. He was diagnosed with aplastic anemia from PVB19 and neutropenia. From June 2013 to January 2019, the patient was admitted 23 times with severe neutropenia and anemia, and on each occasion, PVB19 DNA by PCR was positive. During these multiple admissions, he was treated with antibiotics for neutropenic fever, methicillin-resistant *Staphylococcus aureus* (MRSA) bacteremia, and methicillin-sensitive *Staphylococcus aureus* (MSSA) skin abscesses. On each occasion, he required multiple blood transfusions, and he received intravenous immunoglobulin (IVIG) that resulted in significant improvement of absolute neutrophil count (ANC) count. He had bone biopsy twice, which showed normal maturation of the myeloid series and reduced erythroid progenitor cells consistent with PVB19 infection. PVB19 DNA by PCR remains positive to date. Immunocompromised individuals usually develop a chronic infection from PVB19, and rarely infection with PVB19 can cause severe neutropenia that can be long-lasting and life-threatening. Early recognition and treatment with IVIG are the key to improve the clinical outcome.

## Introduction

Parvovirus B19 (PVB19) is a deoxyribonucleic acid (DNA) virus, the only member of the Parvoviridae family known to be pathogenic in humans [1]. The virus has a direct cytopathic effect on erythroid progenitor cells, resulting in an arrest of hematopoiesis and subsequent anemia. Less frequently, other hematologic abnormalities such as neutropenia and thrombocytopenia have been reported with PVB19 infection [2]. We report a rare case of recurrent neutropenia and anemia due to PVB19 infection in a human immunodeficiency virus (HIV) patient that led to 23 admissions over a period of six years.

## Case presentation

Our patient is a 51-year-old Hispanic male who presented to the Emergency Department (ED) with the complaint of generalized weakness, intermittent dizziness, and headache for two weeks. His medical history was significant for HIV, well-controlled with antiretroviral therapy. He had no history of recent travel or sick contacts. In the ED the patient was hypotensive, tachycardia, and pale. The rest of the physical exam was unremarkable. There was no evidence of active bleeding.

His laboratory tests were significant for severe anemia and neutropenia: hemoglobin 4.4 g/dl, hematocrit 12.4%, main corpuscular volume 89 fL; reticulocyte 0.1%, platelets 261 k/µl, white blood count 2.3 k/µl, and absolute neutrophil count (ANC) 1200 k/µl. Kidney and liver function tests were within normal limits. Peripheral smear did not show any schistocytes, and the hemoglobin electrophoresis showed no hemoglobinopathy. The HIV VL was undetectable. His baseline hemoglobin was 11.7 g/dl, hematocrit 34.5%, platelet count 158 k/µl, white blood count 3.0 k/µl, and ANC 2000 k/µl.

PVB19 DNA by PCR was reported positive; PVB19 immunoglobulin M (IgM) and IgG were reported negative. The patient received intravenous fluids and blood transfusions. Gastroenterology was consulted, and the patient underwent upper endoscopy, colonoscopy, and capsule endoscopy, which did not show any evidence of bleeding. He was discharged with close follow-up.

**Figure 1:**
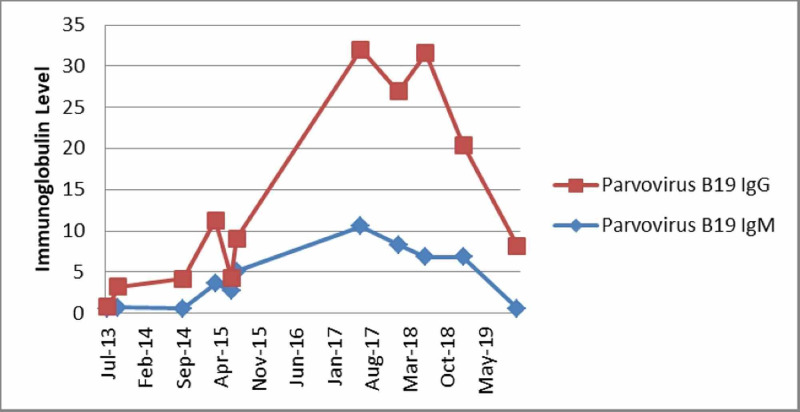
Trend of immunoglobulin throughout multiple admissions Ig: Immunoglobulin

Two months later, the patient returned to the ED with complaints of generalized weakness and shortness of breath. His laboratory tests were significant for severe anemia (hemoglobin 3.4 gm/dl, reticulocyte 0.1%) and severe neutropenia (ANC 200 k/µl). There was no evidence of active bleeding or hemolysis. Serology for PVB19 was repeated, IgM was reported 2.5 (positive), and IgG was 0.7 (negative). PVB19 DNA by PCR was positive. He received a blood transfusion and was started on intravenous Ig 1 gm/kg for a total of five days. His anemia and neutropenia improved, and the patient was discharged. In the past six years, the patient was admitted 23 times with severe neutropenia and anemia, and on each occasion, PVB19 DNA by PCR was positive. He had bone biopsy two times, which showed 50% cellularity with normal maturation of the myeloid series, severe erythroid depletion, and reduced erythroid progenitor cells. The erythroblast showed prominent megaloblastic and giant fibroblastic changes consisted of PVB19 infection. On each admission, he required multiple blood transfusions, and he received intravenous immunoglobulin (IVIG) 1 gm/kg with an average duration of five days. The patient’s PVB19 IgG and IgM trends throughout his multiple admissions are presented in Figure 1.

**Figure 2:**
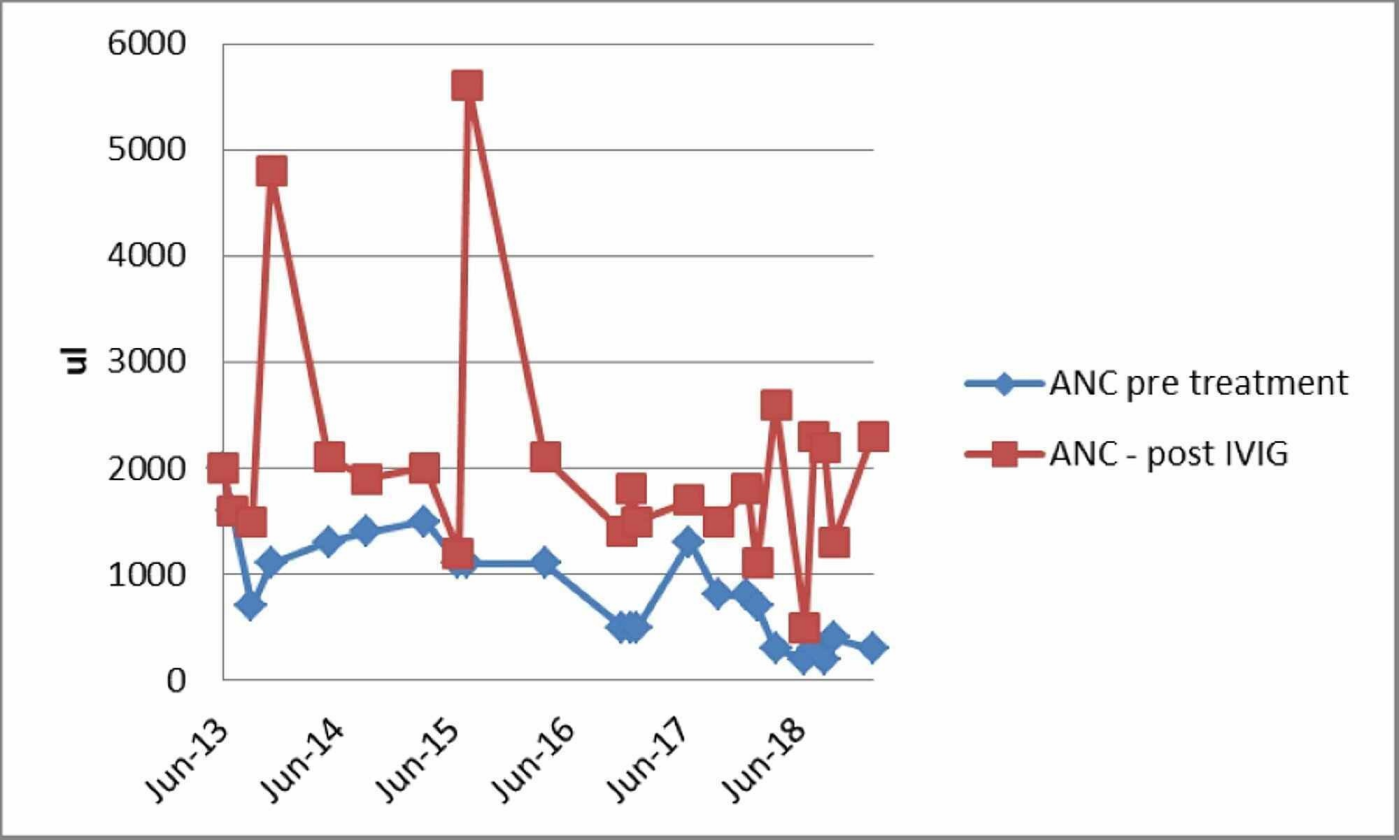
Trend of absolute neutrophil count on admission and post intravenous immunoglobulin throughout his multiple admissions ANC: Absolute neutrophil count; IVIG: intravenous immunoglobulin

The patient’s ANC on admission and post intravenous immunoglobulin throughout his multiple admissions are presented in Figure 2.

## Discussion

PVB19 is a DNA virus, the only member of the Parvoviridae family known to be pathogenic in humans. PVB19 is transmitted mainly via droplets, but also vertically through the placenta and through blood transfusion. Certain groups, such as pregnant women, immunocompromised patients, and individuals with chronic hemolytic anemia, are at high risk of acquiring the PVB19 infection.

Acute infection is associated with a viremic phase, shortly followed by IgM production (10-14 days after the infection) and later by the production of the IgG antibody against the viral capsid. Viremia declines with IgM production. An IgM level decreases after a few months, but IgG persists longer and confers lasting protection against reinfection [3]. Serum from patients with persistent infection lacks antibodies to PVB19 or contains low levels of non-neutralizing IgM or IgG antibodies [4]. In our case, detectable PVB19 IgM first appeared after two months, and detectable PVB19 IgG was first reported positive after 20 months.

The clinical manifestations of the infection with PVB19 vary with age, hematologic as well as the immunologic status of the host. Immunocompromised individuals are unable to mount an adequate immune response, resulting in chronic infection and anemia [1]. Neutropenia has been reported in immunocompromised individuals with PVB19 infection and usually improves after the administration of intravenous immunoglobulin [4]. Several mechanisms have been proposed for the neutropenia, which includes hemophagocytic syndrome, bone marrow hypoplasia, the inhibitory effect of PVB19 on granulocyte-forming units, leukocyte elimination or consumption, and chronic anti-neutrophilic antibodies [5-7]. The exact mechanism of neutropenia is unknown in our case. Our patient underwent bone marrow biopsy twice; the myeloid lineage showed no hypoplasia or abnormalities in the maturation, and there was no evidence of hemophagocytic syndrome. Neutropenia is commonly seen in HIV-infected patients usually in the late stages of HIV but sometimes during the early stages of the infection. In HIV-induced neutropenia, bone marrow biopsy shows myelodysplasia with a left shift of the myeloid series and abnormalities in maturation of the cells along the granulocyte lineage, which were not seen in our patient [8]. The patient was adherent to his antiretroviral therapy and had a persistently suppressed HIV viral load. Furthermore, his neutropenia improved on each occasion after the administration of IVIG, supporting our hypothesis that it was caused by the infection from PVB19.

## Conclusions

Immunocompromised individuals can develop a chronic infection from PVB19, resulting in chronic aplastic anemia and less frequently severe neutropenia that can be long-lasting and life-threatening. Early recognition and treatment with IVIG are the key in order to improve the clinical outcomes.
